# Solute Carrier Family 35 A2 (SLC35A2) Promotes Tumor Progression through MYC-Mediated Pathways in Colorectal Cancer

**DOI:** 10.7150/ijms.109767

**Published:** 2025-03-29

**Authors:** Kuei-Yen Tsai, Po-Li Wei, Cheng-Chin Lee, Crystal Ngofi Zumbi, G. M. Shazzad Hossain Prince, Uyanga Batzorig, Chien-Yu Huang, Yu-Jia Chang

**Affiliations:** 1 Graduate Institute of Clinical Medicine, College of Medicine, Taipei Medical University, Taipei 11031, Taiwan.; 2 Department of Surgery, School of Medicine, College of Medicine, Taipei Medical University, Taipei 11031, Taiwan.; 3 Division of General Surgery, Department of Surgery, Shuang Ho Hospital, Taipei Medical University, New Taipei City 235041, Taiwan.; 4 Division of Colorectal Surgery, Department of Surgery, Taipei Medical University Hospital, Taipei Medical University, Taipei 11031, Taiwan.; 5 Cancer Research Center and Translational Laboratory, Department of Medical Research, Taipei Medical University Hospital, Taipei Medical University, Taipei 11031, Taiwan.; 6 Graduate Institute of Cancer Biology and Drug Discovery, Taipei Medical University, Taipei 11031, Taiwan.; 7 Department of Dermatology, University of California, San Diego, La Jolla, CA 92093, USA.; 8 School of Medicine, National Tsing Hua University, Hsinchu 300044, Taiwan.; 9 Institute of Molecular and Cellular Biology, National Tsing Hua University 300044, Hsinchu, Taiwan.; 10 Department of Pathology, Wan Fang Hospital, Taipei Medical University, Taipei, Taiwan.; 11 Cell Physiology and Molecular Image Research Center, Wan Fang Hospital, Taipei Medical University, Taipei, Taiwan.; 12 Graduate Institue of Medical Sciences, National Defense Medical Center, Taipei 114201, Taiwan

**Keywords:** biomarker, colorectal cancer, nucleotide sugar transporters, SLC35A family, SLC35A2, metastasis, proliferation, chemoresponse, immunomodulatory

## Abstract

Colorectal cancer (CRC) is one of the most prevalent cancers, posing a significant threat to human life. Although therapeutic approaches for advanced-stage patients have improved in recent years, there is still room for enhancing treatment response. Recent evidence suggests that dysregulation of nucleotide sugar transporters (NSTs) is associated with the development and progression of tumors. Therefore, this study aims to explore the potential therapeutic and prognostic implications of the solute carrier family 35 A (SLC35A) members in CRC. To achieve this, we performed integrative bioinformatics analysis using various publicly available databases, including GENT2, TCGA, UALCAN, cBioPortal, Kaplan-Meier plotter, The ROC plotter, GDSC, TISIDB, and TIMER. We compared gene expression profiles between CRC tumors and adjacent normal tissues, revealing that only SLC35A2 exhibited significant upregulation in tumors, while the other family members were downregulated. Additionally, higher SLC35A2 expression was found in microsatellite stable (MSS) colorectal tumors. Further analysis of TCGA and GEO datasets showed that patients with high SLC35A2 expression experienced poorer relapse-free survival. Next, we conducted gene set enrichment analysis (GSEA), and the results indicated that the upregulation of SLC35A2 is linked to cellular metabolism pathways, such as MYC Targets V2, Steroid Biosynthesis, Pentose Phosphate Pathway, and TCA Cycle. Furthermore, our CRC cell models revealed the tumor-promoting role of SLC35A2 and discovered that the upregulation of SLC35A2 is associated with chemoresistance against irinotecan. Additionally, we observed a negative correlation between SLC35A2 expression and the infiltration of immune cells, particularly cytotoxic CD8+ T cells and B cells. This suggests the immunomodulatory role of SLC35A2. In summary, SLC35A2 is abnormally upregulated in CRC, and patients with high SLC35A2 expression tend to have poor relapse-free survival. This may be due to its involvement in regulating cancer cell metabolic reprogramming, promoting tumor progression, modulating the immune landscape, and influencing treatment response. Consequently, SLC35A2 could serve as a significant prognostic factor and a potential therapeutic target in CRC.

## Introduction

Colorectal cancer (CRC) is one of the most common malignancies worldwide, particularly in developed countries 1. According to data from GLOBOCAN 2022, CRC ranks as the third most common cancer globally and the second leading cause of cancer-related deaths. Each year, nearly two million individuals are diagnosed with CRC, and approximately one million succumb to it 2. Early in tumor development, genomic instability emerges as a crucial molecular event contributing to CRC 3. However, the precise mechanisms driving the development of CRC remain elusive. Multiple genes and a multistep process have been implicated in the pathogenesis of CRC, as highlighted in various studies 4. Despite notable advancements in chemotherapy and targeted therapies in recent times, the 5-year survival rate for patients with advanced CRC remains unsatisfactory 5. Consequently, there is an urgent and pressing need for innovative treatment strategies that can enhance patient outcomes.

Humans have approximately 400 different solute carriers (SLCs), which are classified into 65 families based on their sequence homology and function. SLCs are crucial in transporting various solutes across biological membranes, including inorganic ions, amino acids, lipids, sugars, neurotransmitters, and drugs 6. Given the escalated energy and nutrient requirements of tumor cells, specific SLCs are upregulated in these cells. Consequently, inhibiting these transporters and disrupting the supply of energy sources or nutrients could potentially impede tumor progression 7. Furthermore, manipulating these transporters might alter the response of tumor cells to chemotherapy drugs, making the study of SLCs an intriguing area of research.

The SLC35 family, which encodes proteins, consists of a highly conserved group of type III transmembrane proteins. It is further divided into seven subfamilies (SLC35A-SLC35G) based on the transported substrates 8. The SLC35 family, known as nucleotide sugar transporters (NSTs), plays a crucial role in transporting nucleotide sugars and 3'-phosphoadenosine-5'-phosphosulfate (PAPS) from the cytosol to the endoplasmic reticulum and Golgi apparatus. These transported molecules serve as substrates for glycosylation and sulfation processes involved in modifying proteins, lipids, and proteoglycans. The transport function of NSTs is essential for glycosylation modifications and biological development. For instance, mutations in two NST genes, SLC35A1 and SLC35C1, have been associated with a congenital disorder of glycosylation II (CDG II) 9.

The SLC35A family genes belong to a subfamily of SLC35, consisting of five members designated as SLC35A1-5. While they are all involved in nucleotide sugar transport, they exhibit slight differences in substrate specificity, transport rates, or involvement in specific biosynthetic pathways 10. These variations can be attributed to variances in gene sequences, protein structures, and expression patterns among the family members, potentially contributing to the diversity and specificity of their cellular functions and physiological processes. It is important to highlight that although the SLC35A family members are known to play a significant role in protein glycosylation, further investigation is necessary to explore their interactions with other SLCs and their potential involvement in biological functions 11, 12.

Pertaining to cancer, a previous report identified that high expression of SLC35A2 is corresponded with worse recurrence-free survival in patients with breast cancer 13. The role of SLC35A2 in regulating glucosylation was revealed promoting metastasis in hepatocellular carcinoma (HCC) 14. In a human pan-cancer bioinformatic analysis also unravelled SLC35A2 as a potential prognosis biomarker including colon adenocarcinoma 15. However, the mechanism of SLC35A2 in regulating CRC is not yet discoved. To decipher and acquire cancer hallmarks necessitate the integration of diverse omics disciplines, such as genomics, epigenomics, transcriptomics, and proteomics 16. RNA sequencing has also emerged as a vital method for transcriptome analysis, with significant advancements being made in recent years. These techniques have been extensively applied in various facets of cancer research, including the identification of biomarkers, assessment of cancer heterogeneity, examination of drug resistance, investigation of the cancer immune microenvironment, exploration of immune therapy, and evaluation of cancer neoantigens 17. The utilization of high-throughput omics technologies has generated vast amounts of data, enabling a comprehensive and extensive exploration of the potential roles played by specific gene families in cancer 18, 19.

In our study, we conducted comprehensive multi-omics analyses to investigate the expression patterns of SLC35A family genes in human CRC. To accomplish this, we utilized various platforms such as Gene Expression database of Normal and Tumor tissues 2 (GENT2), cBioPortal, and The University of Alabama at Birmingham cancer data analysis portal (UALCAN) to assess the expression levels, mutations, and epigenetic alterations of SLC35A family genes. Moreover, we employed the Kaplan-Meier Plotter and The Cancer Genome Atlas (TCGA) data analysis to examine the correlation between these SLC35A members and clinical outcomes in CRC. To further understand the potential biological pathways involving SLC35A2 in colorectal cancer, we utilized gene set enrichment analysis (GSEA) and explored the relationship between SLC35A2 expression and drug sensitivity by analyzing data from Cancer Cell Line Encyclopedia (CCLE) and TCGA. Additionally, we validated the results using CRC cell models. Furthermore, using the Tumor Immune Estimation Resource (TIMER) database, we identified potential biomarkers related to immune infiltration. Our study reveals that SLC35A2 is significantly upregulated in CRC and is associated with patient prognosis and sensitivity to the drug irinotecan. Thus, SLC35A2 holds promise as a potential biomarker for the diagnosis and treatment of CRC.

## Methods and materials

### Analysis of gene expression levels

We used GENT2 (http://gent2.appex.kr/gent2/), which is a web portal to perform analyses of The Gene Expression Omnibus (GEO) gene expression data 20 to investigate the expression of the SLC35A family members across tumor and adjacent normal tissues. In addition, the RNA-seq data of the TCGA COAD (colon adenocarcinoma) project, processed through the STAR workflow, was obtained from the TCGA database (https://portal.gdc.cancer.gov). After normalization using the log_2_ transformation with the addition of 1, we compared the expression of the SLC35A family between tumor and normal tissues, as well as assessed the expression correlation among each member of the family. The statistical analysis was performed using the R packages stats [4.2.1] and car [3.1-0], employing the Wilcoxon rank sum test. Finally, the results were visualized using the ggplot2 [3.3.6] package.

### Analysis of DNA methylation and genomic alterations

UALCAN (https://ualcan.path.uab.edu/) is an interactive web resource for analyzing cancer OMICS data, featuring high-quality graphics and user-friendly interfaces. It specifically focuses on evaluating the impact of promoter methylation on gene expression for epigenetic regulation 21. We used UALCAN to analyze methylation levels of SLC35A family gene promoters in CRC and adjacent normal tissues. We performed an analysis of the mutation status of SLC35A family genes using the CRC dataset (TCGA, Firehose Legacy) from cBioPortal (http://cbioportal.org) 22.

### Survival analysis

The Kaplan-Meier plotter (https://kmplot.com/analysis/index.php?p=background) can be used to estimate the correlation between gene expression and survival in 21 tumor types 23. We used this online tool to evaluate the prognostic value of the SLC35A family in CRC patients from GEO database. Patient samples were split into two groups based on the median expression level of the mRNA and we calculated the log-rank P value, hazard ratio (HR), and 95 % confidence interval (95% CI). Moreover, the RNA seq data of COAD (FPKM) processed through the STAR workflow and clinical information were obtained from the TCGA database (https://portal.gdc.cancer.gov). The survival data of patients was extracted from a literature 24. Cox regression and Kaplan-Meier analyses were conducted utilizing the survival [3.3.1] package. Results were visualized using the survminer and ggplot2 packages. Statistical significance was defined as a P-value less than 0.05.

### Functional enrichment analysis

We utilized the DESeq2 [1.36.0] package to perform differential gene expression analysis between the high and low expression groups of SLC35A2 in TCGA COAD data 25. The differential expression genes (DEGs) were filtered using a threshold of adjusted logFC ≥ 1 and P value ≤ 0.05. In addition, we used the CRC dataset (TCGA, Firehose Legacy) from cBioPortal to perform Spearman correlation analysis to identify co-expression genes of SLC35A2. Gene Set Enrichment Analysis (GSEA) was performed using the clusterProfiler [4.4.4] package 26. The generated results were then visualized using the ggplot2 [3.3.6] package.

### Quantitative reverse transcription polymerase chain reaction (qRT-PCR)

Total RNA was isolated from cultured cells using TRIZOL reagent (Invitrogen Life Technologies, Carlsbad, CA, USA), following standard protocols. For cDNA synthesis, 8 μg of total RNA was utilized in a 20-μL reaction volume, employing a cDNA Synthesis Kit (Invitrogen Life Technologies). Reverse transcription was carried out according to the manufacturer's instructions. Real-time PCR was subsequently conducted with ABI SYBR Green Master Mix (Applied Biosystems), using the ABI 7500 FAST system for thermal cycling. Each sample was analyzed in triplicate, and the entire experiment was repeated three times. Gene expression levels were normalized to the housekeeping gene glyceraldehyde 3-phosphate dehydrogenase (GAPDH) for accurate quantification.

### Chemicals, reagents, and cell culture

Human colorectal cancer (CRC) cell lines, DLD-1 (CCL-221) and HCT 116 (CCL-247), were sourced from the American Type Culture Collection (ATCC, Rockville, MD, USA). The cells were maintained in RPMI 1640 medium, supplemented with 10% fetal bovine serum (FBS) (SAFC Biosciences, Lenexa, KS, USA) and 1% penicillin-streptomycin (100 IU/mL penicillin and 100 μg/mL streptomycin), under standard conditions at 37°C in a humidified incubator with 5% CO₂. Cells were sub-cultured or used for experiments before reaching 80% confluence. The chemotherapeutic agent SN-38 (Selleck, Houston, TX, USA) was dissolved in dimethyl sulfoxide (DMSO) to a concentration of 10 mM and subsequently diluted with RPMI 1640 medium containing 10% FBS for experimental use.

### SLC35A2 silencing in cell lines

For the knockdown (KD) of SLC35A2, short hairpin RNA (shRNA; TRCN0000043548) targeting human SLC35A2 (NM_005660) was acquired from the National RNAi Core Facility at Academia Sinica, Taiwan. DLD-1 and HCT 116 cells were transfected with either SLC35A2-specific shRNA or non-target control shRNA. Stably transfected cells were selected with puromycin over a two-week period. The efficiency of SLC35A2 silencing was confirmed by quantitative reverse transcription polymerase chain reaction (RT-qPCR).

### Examination of cell proliferation and viability

Cell proliferation was initially monitored using the xCELLigence system. Colorectal cancer cells were seeded at a density of 0.6 × 10⁴ cells per well into the bottom chambers of tissue culture E-Plates. Cell growth was continuously tracked through cell index measurements until saturation was reached. For the sulforhodamine B (SRB) assay, CRC cells with SLC35A2 knockdown and scrambled control conditions were seeded into 96-well plates at the same density (0.6 × 10⁴ cells per well) and incubated overnight at 37°C in a humidified incubator with 5% CO₂. Following incubation, cells were fixed overnight with 10% (wt/vol) trichloroacetic acid at 4°C, and then stained with 0.4% (wt/vol) sulforhodamine B for 30 minutes at room temperature. The stained cells were washed twice with 1% acetic acid, air-dried overnight, and the protein-bound dye was solubilized in 10 mM Tris base solution. Optical density (OD) was measured at 515 nm using a microplate reader (Bio-Rad Laboratories, Hercules, CA, USA). Scrambled control cells were used as the baseline, and fold changes in cell proliferation were calculated by comparing the OD values of SLC35A2 knockdown cells to this baseline.

### Wound healing and transwell migration assay

The migratory activity of scrambled control and SLC35A2 knockdown cells was assessed in real-time using the Luma System. A suspension of 5 × 10⁵ cells/mL was prepared, and 70 μL of this suspension was seeded into Ibidi culture inserts (Ibidi GmbH, Münich, Germany) placed in the center of a 35 mm dish. After overnight incubation at 37°C, the inserts were removed, and 3 mL of fresh medium was added to the dish. The dish was then transferred to a stage-top incubator on an inverted microscope, and cell migration into the cell-free gap was monitored using a 20x objective lens. For the transwell migration assay, BD Falcon cell culture inserts were utilized. Aliquots of 1 × 10⁵ CRC cells suspended in 500 μL of serum-free RPMI medium were seeded into the upper chambers, while the lower chambers were filled with 1 mL of RPMI medium supplemented with 10% fetal bovine serum and 1% penicillin-streptomycin. After 48 hours of incubation at 37°C in a 5% CO₂ environment, each well and chamber was washed once with 1 mL of 1× PBS. The cells were then fixed with a methyl alcohol solution briefly, and non-migrated cells from the upper chamber were carefully removed using cotton swabs. The cells that had migrated to the reverse side of the membrane were stained with 0.1% crystal violet and incubated at room temperature for 8 hours. Following staining, the crystal violet was removed, and the number of migrated cells was counted under a microscope (Olympus IX, Olympus, Tokyo, Japan) at 10x magnification using a handheld cell counter.

### Chemotherapeutic response analysis

The ROC plotter (https://www.rocplot.org/) is a web tool that integrates gene expression data and therapy response information in breast, ovarian, and colorectal cancer patients, as well as glioblastomas 27. We utilized the tool to assess the sensitivity and specificity of SLC35A2 as a classifier for determining chemotherapy responsiveness in CRC patients. The tool generated a box plot to visualize the expression levels of SLC35A2 in both responders and non-responders. Additionally, it calculated the area under the curve (AUC) and corresponding p-values. The statistical significance of the observed differences was evaluated using the Mann-Whitney U test. The prediction of chemotherapy response for each CRC sample from TCGA data was performed using the R package pRRophetic based on the Genomics of Drug Sensitivity in Cancer (GDSC) database (https://www.cancerrxgene.org/) 28. The ridge regression was utilized to estimate the half-maximal inhibitory concentration (IC_50_) of the samples.

### Analysis of tumor-infiltrating immune cells

The TIMER (https://cistrome.shinyapps.io/timer/) and TISIDB (Tumor-Immune System Interaction Database; https://cis.hku.hk/TISIDB/) are web tools for conducting analysis of immune infiltrates in various cancer types. The abundances of six immune infiltrates, including B cells, CD4+ T cells, CD8+ T cells, neutrophils, macrophages, and dendritic cells, were estimated using the TIMER algorithm 29. We utilized the TIMER and TISIDB to assess the association between genes and the infiltration of immune cells in CRC patients 30.

## Results

### Transcriptional expression levels of SLC35A family in different types of cancer

**Figure 1:**
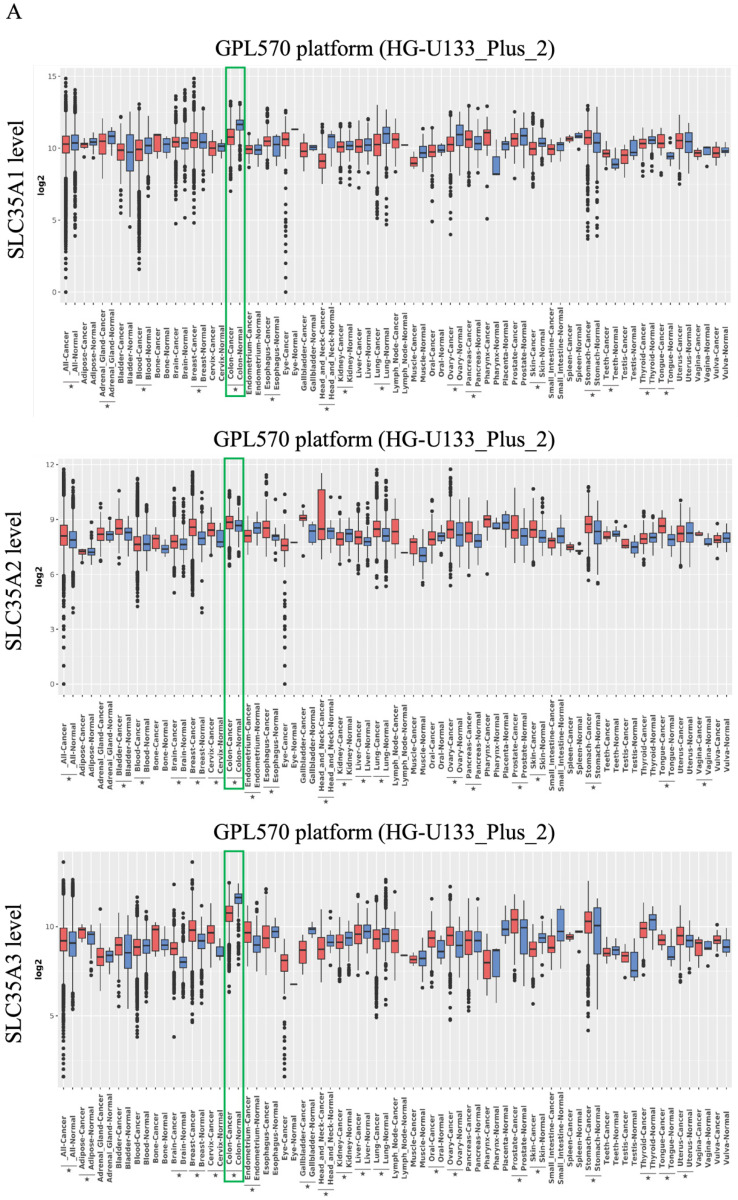

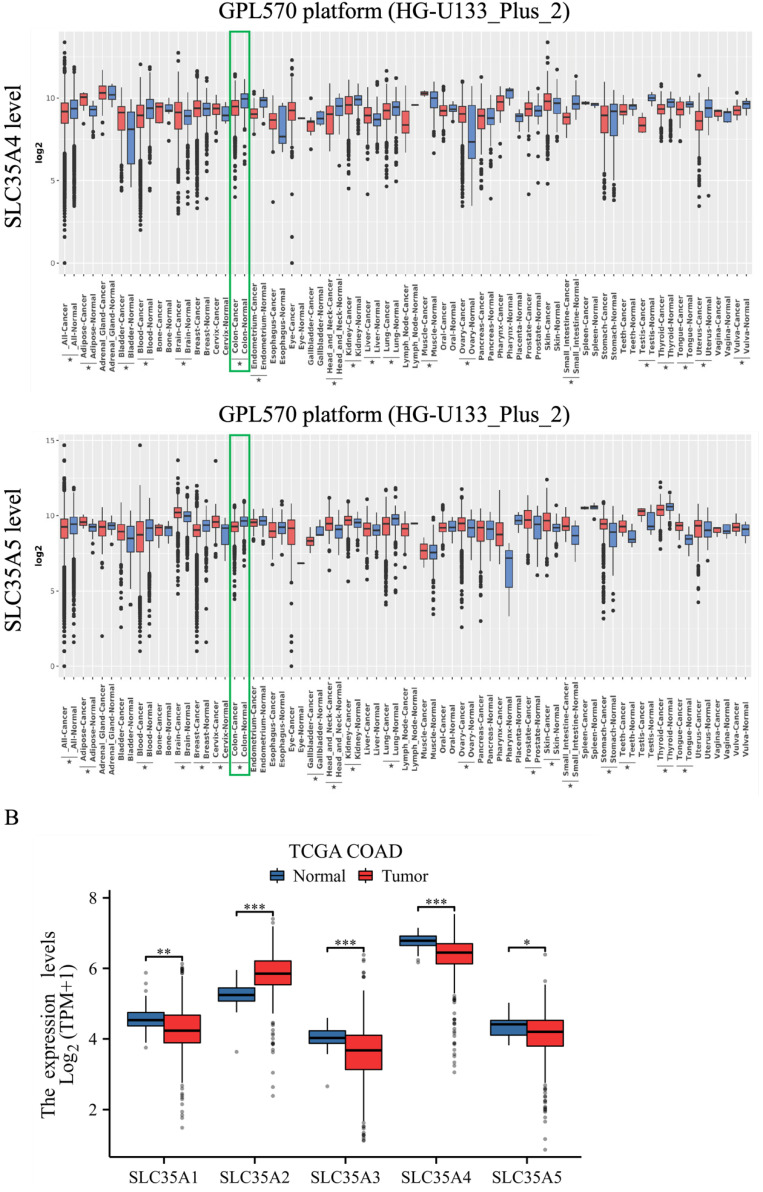
** Overview of mRNA expression levels of SLC35A1-5 in multiple types of cancer.** (A) The analysis compared expressions of genes in tumor tissues relative to corresponding normal tissues from the GENT2 database. A two-sample T-test was used, and asterisks indicated p-values less than 0.05. (B) The mRNA expression of SLC35A1-5 between tumor and normal tissues in CRC based on TCGA COAD data. *p < 0.05, ** p < 0.01, *** p < 0.001.

To investigate the expression patterns of the SLC35A family, we utilized the GENT2 database, as shown in Figure 1A. Our analysis revealed observations for each family member in various tumor and non-tumor tissues. SLC35A1 demonstrated lower expression in tumor tissues compared to normal tissues, including CRC. Nevertheless, in other cancers related to the digestive system, such as the tongue, esophagus, stomach, and pancreas, SLC35A1 exhibited higher expression in tumor tissues. SLC35A2, on the other hand, generally exhibited higher expression in tumor tissues compared to normal tissues, including CRC. However, in specific tissues like the kidney, endometrium, and blood, SLC35A2 displayed lower expression. SLC35A3 showed high expression in multiple tumor tissues but lower expression in some common cancers, such as colon, lung, and liver. Both SLC35A4 and SLC35A5 consistently exhibited lower expression in tumor tissues across all tissue types, including colorectal cancer. Besides, SLC35A2 is highly expressed in advance stages of CRC, and patients with microsatellite stable (MSS) CRC exhibits significantly higher level of SLC35A2 than the patients with microsatellite instable (MSI) CRC (Supplementary Figure 1).

To validate the expression patterns of SLC35A family genes in patients with CRC, we utilized the TCGA COAD data, as shown in Figure 1B. The results confirmed that SLC35A2 exhibited higher expression in tumor tissues compared to normal tissues. In contrast, the other family members, including SLC35A1, SLC35A3, SLC35A4, and SLC35A5, demonstrated lower expression in tumor tissues. These findings suggest a potential role for SLC35A2 in the tumorigenesis of CRC.

### Methylation, mutation, and correlation analysis of SLC35A family in TCGA COAD

**Figure 2:**
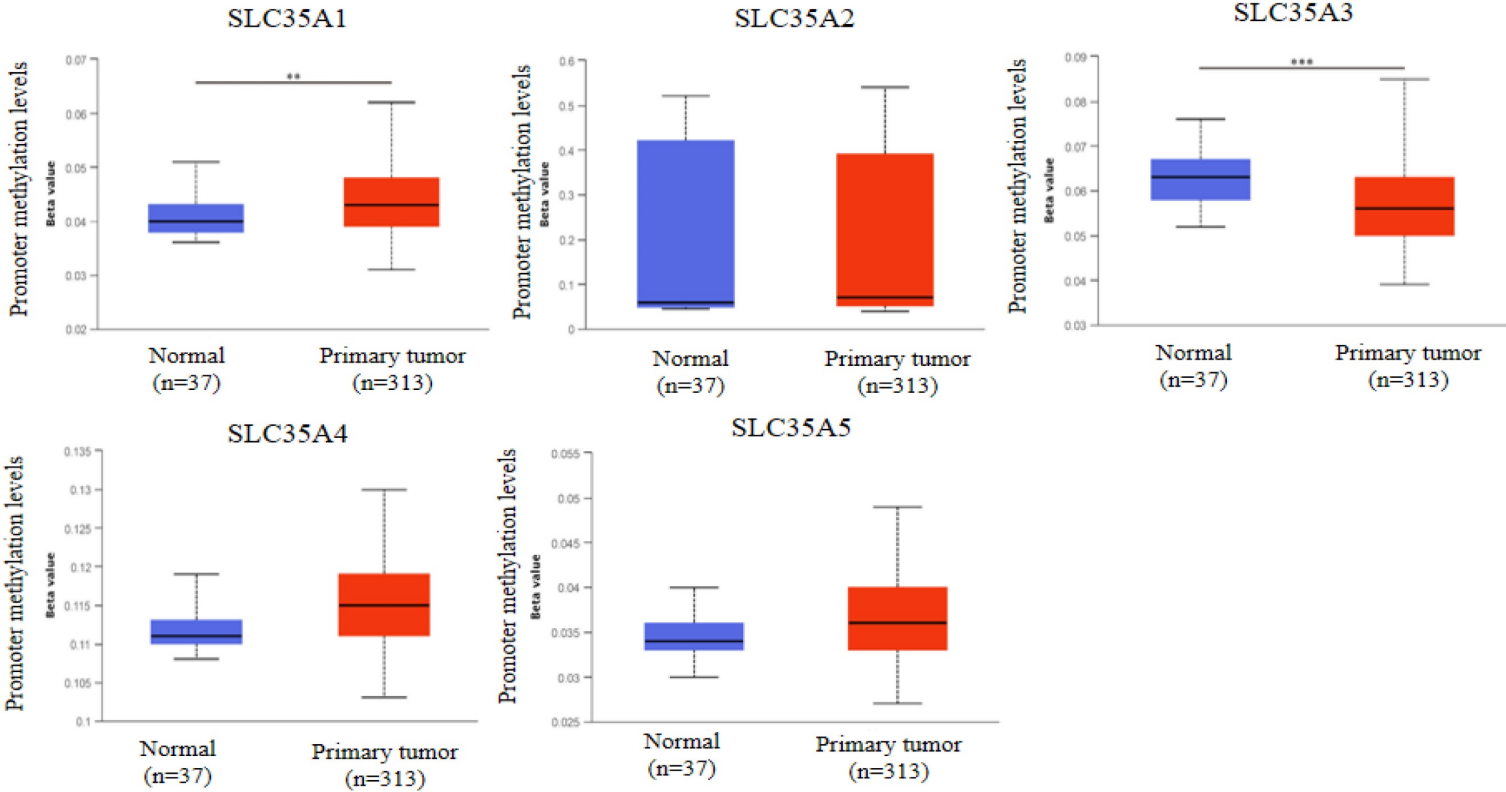
** Promoter methylation analysis of SLC35A family members in CRC.** Methylation levels were compared between normal and tumor tissues. * p < 0.05, ** p < 0.01, *** p < 0.001.

**Figure 3:**
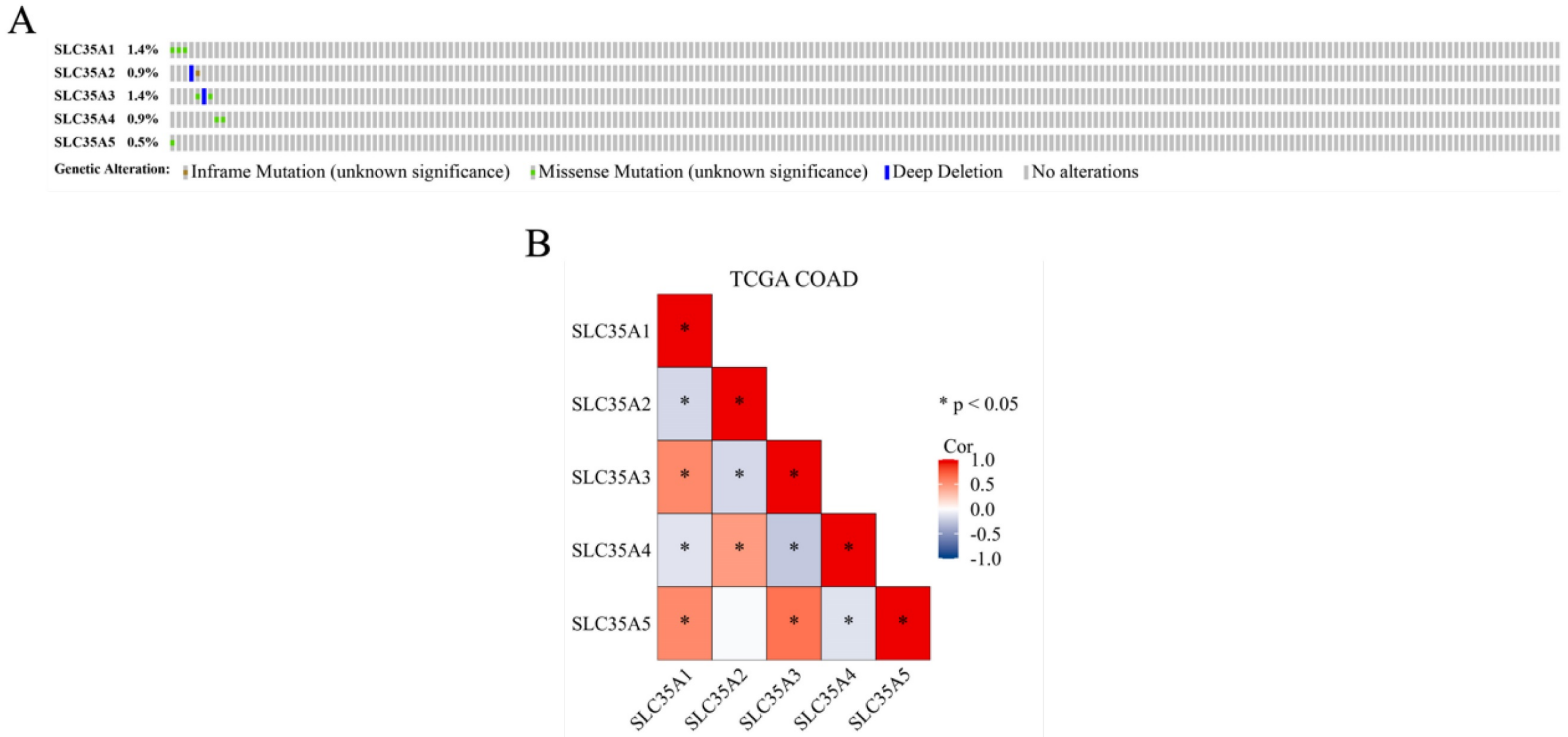
** Genomic alterations and correlation analysis of SLC35A family genes in CRC.** (A) The OncoPrint schematic showed alteration details of the SLC35A family in TCGA Firehose Legacy dataset. (B) Pearson's rank correlation was applied to TCGA COAD data to determine the correlation between genes in the SLC35A family.

DNA methylation, an epigenetic modification known for its regulatory role in gene expression, plays a crucial role in the development and progression of cancer 31. In our study, we investigated the methylation status of the SLC35A family promoters in CRC using the UALCAN database (Figure 2). By comparing methylation levels between tumor and normal tissues, we observed a higher methylation level in the SLC35A1 promoter in tumor tissues. Conversely, SLC35A3 displayed lower methylation levels in tumor tissues. No significant differences in methylation were observed for the other family members. To explore the mutation landscape of the SLC35A family in CRC, we utilized the cBioPortal tool (Figure 3A). The results indicated that SLC35A1 and SLC35A3 had the highest mutation frequencies (1.4%), predominantly consisting of missense mutations. SLC35A3 also exhibited deep deletions in addition to missense mutations. SLC35A2 displayed two types of mutations: inframe mutations and deep deletions. Missense mutations were predominantly associated with both SLC35A4 and SLC35A5. We further examined the expression correlations among the SLC35A family members in CRC (Figure 3B). The results revealed significant positive correlations between SLC35A1 and SLC35A5 (Coefficient: 0.549) and between SLC35A1 and SLC35A3 (Coefficient: 0.545). Moreover, SLC35A2 exhibited a significant positive correlation with SLC35A4 (Coefficient: 0.464), while SLC35A3 displayed a significant positive correlation with SLC35A5 (Coefficient: 0.646). On the contrary, SLC35A1 showed significant negative correlations with SLC35A2 (Coefficient: -0.184) and SLC35A4 (Coefficient: -0.129). SLC35A2 demonstrated a significant negative correlation with SLC35A3 (Coefficient: -0.179), and SLC35A3 displayed a significant negative correlation with SLC35A4 (Coefficient: -0.257). Additionally, SLC35A4 exhibited a significant negative correlation with SLC35A5 (Coefficient: -0.131).

### Prognostic value of SLC35A family genes in patients with CRC

**Figure 4:**
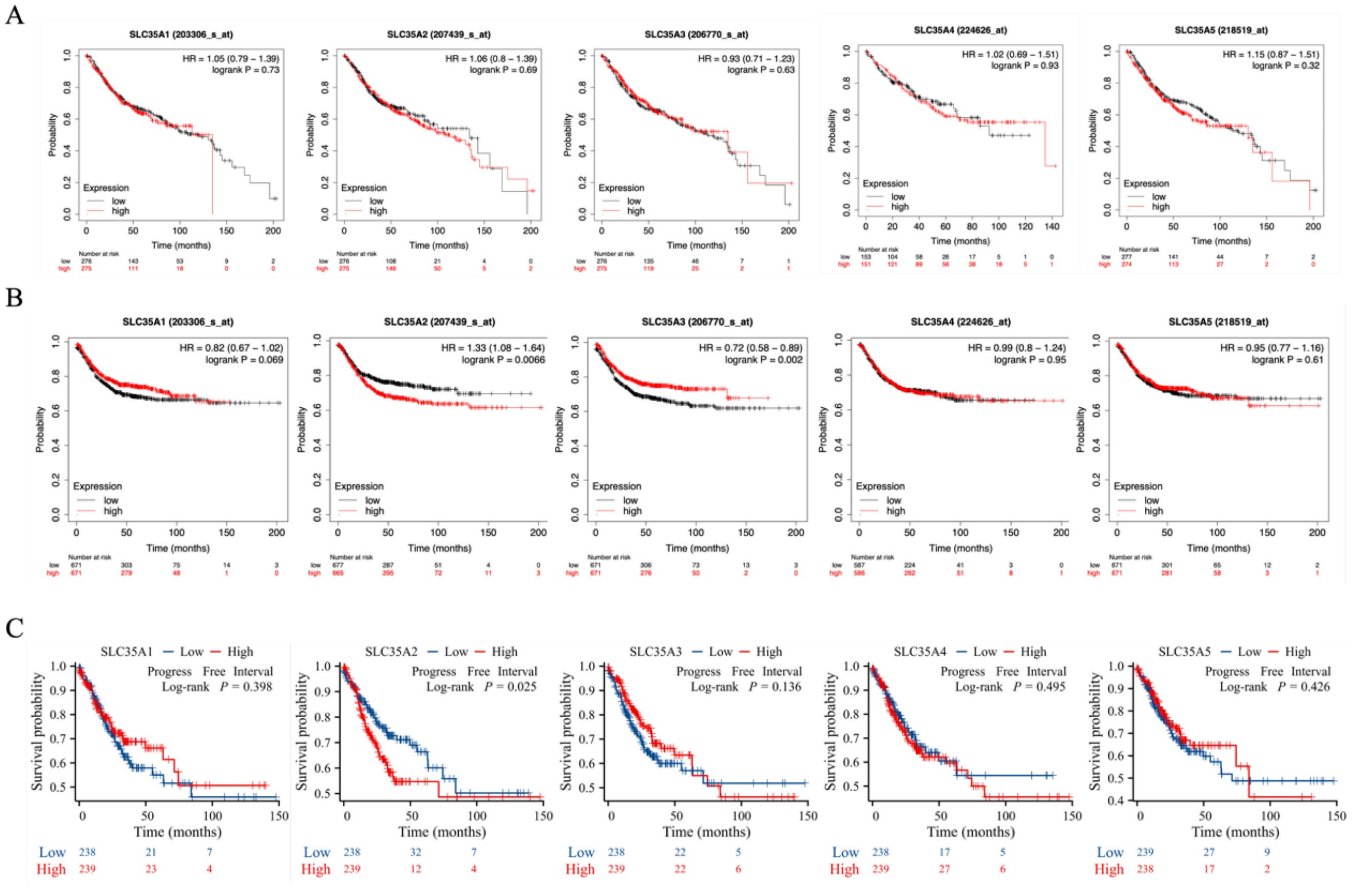
** SLC35A family genes expression with clinical outcome in CRC patients.** (A) overall survival and (B) relapse-free survival analysis based on Kaplan-Meier Plotter database. (C) Kaplan-Meier plots for progression-free interval were analyzed using TCGA COAD data.

In our study, we investigated the correlation between the gene expression of the SLC35A family and the prognosis of CRC patients. Initially, we conducted Kaplan-Meier analysis using gene chip data from the GEO database within the Kaplan-Meier Plotter (Figure 4A and 4B). The analysis revealed that CRC patients with high expression of SLC35A2 had a lower relapse-free survival compared to those with low expression, indicating a poorer outcome for patients with high SLC35A2 expression (hazard ratio (HR) = 1.33, p for trend = 0.0066). Conversely, patients with increased expression of SLC35A3 had a more prolonged relapse-free survival than those with low expression, suggesting a better prognosis for patients with high SLC35A3 expression (hazard ratio (HR) = 0.72, p for trend = 0.002). However, no significant correlation was observed between the gene expression of SLC35A1, SLC35A4, and SLC35A5 and CRC patients' survival.

To validate the findings from the Kaplan-Meier Plotter, we performed Kaplan-Meier analysis using RNA-seq data from the TCGA (Figure 4C). The results consistently demonstrated that patients with high expression of SLC35A2 had a shorter relapse-free interval than those with low expression, corroborating the trends observed in the GEO data. Moreover, no significant correlation was found between the gene expression of the other four family members and patient prognosis.

Subsequently, we conducted an analysis to determine if age, gender, and SLC35A family expression were risk factors for survival in CRC patients (Table 1). Univariate and multivariate Cox analyses based on the progression-free interval indicated that high expression of SLC35A2 was an independent risk factor for survival (p = 0.026).

### Exploration of SLC35A2 functional pathways in CRC

**Figure 5:**
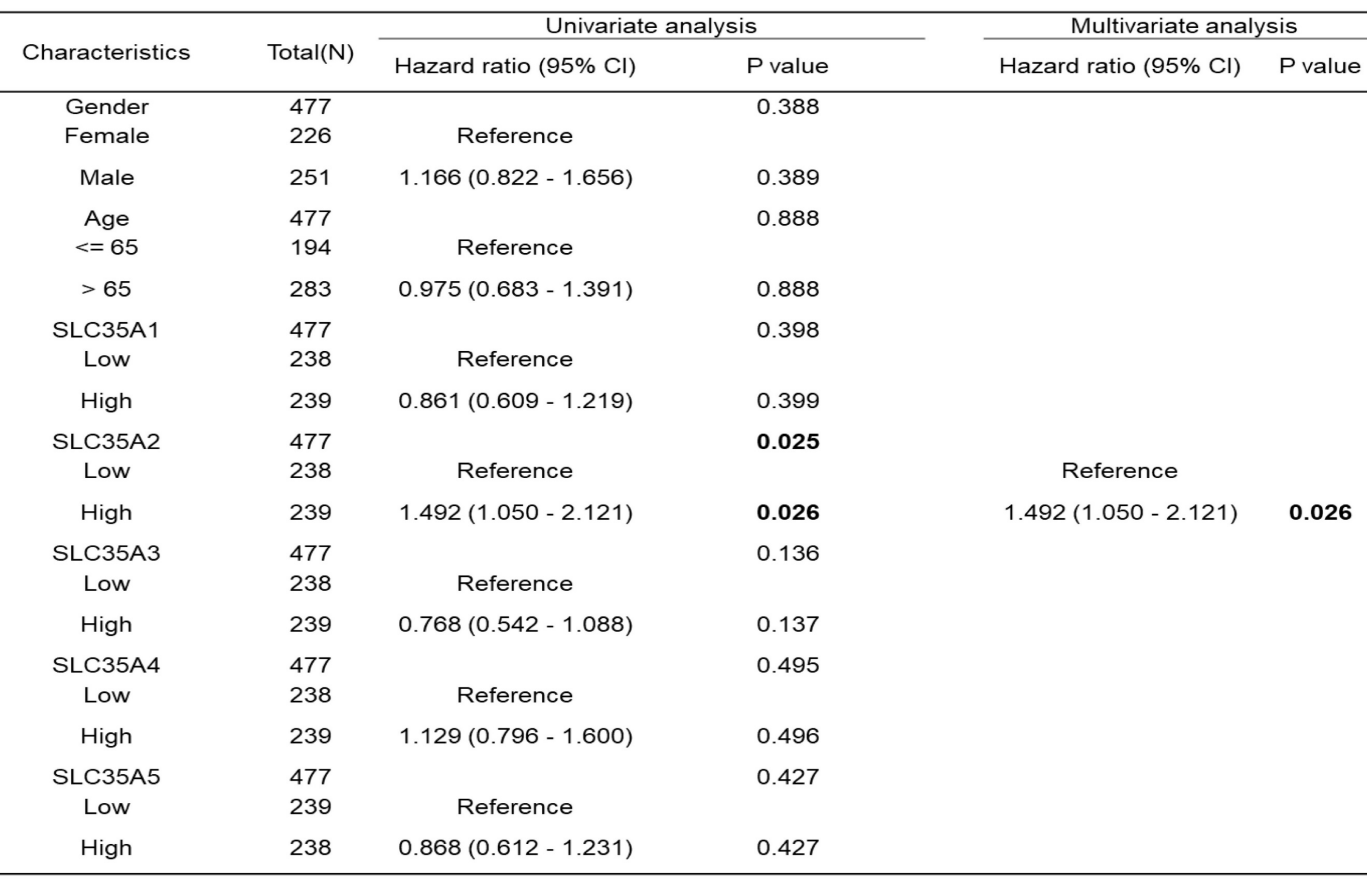

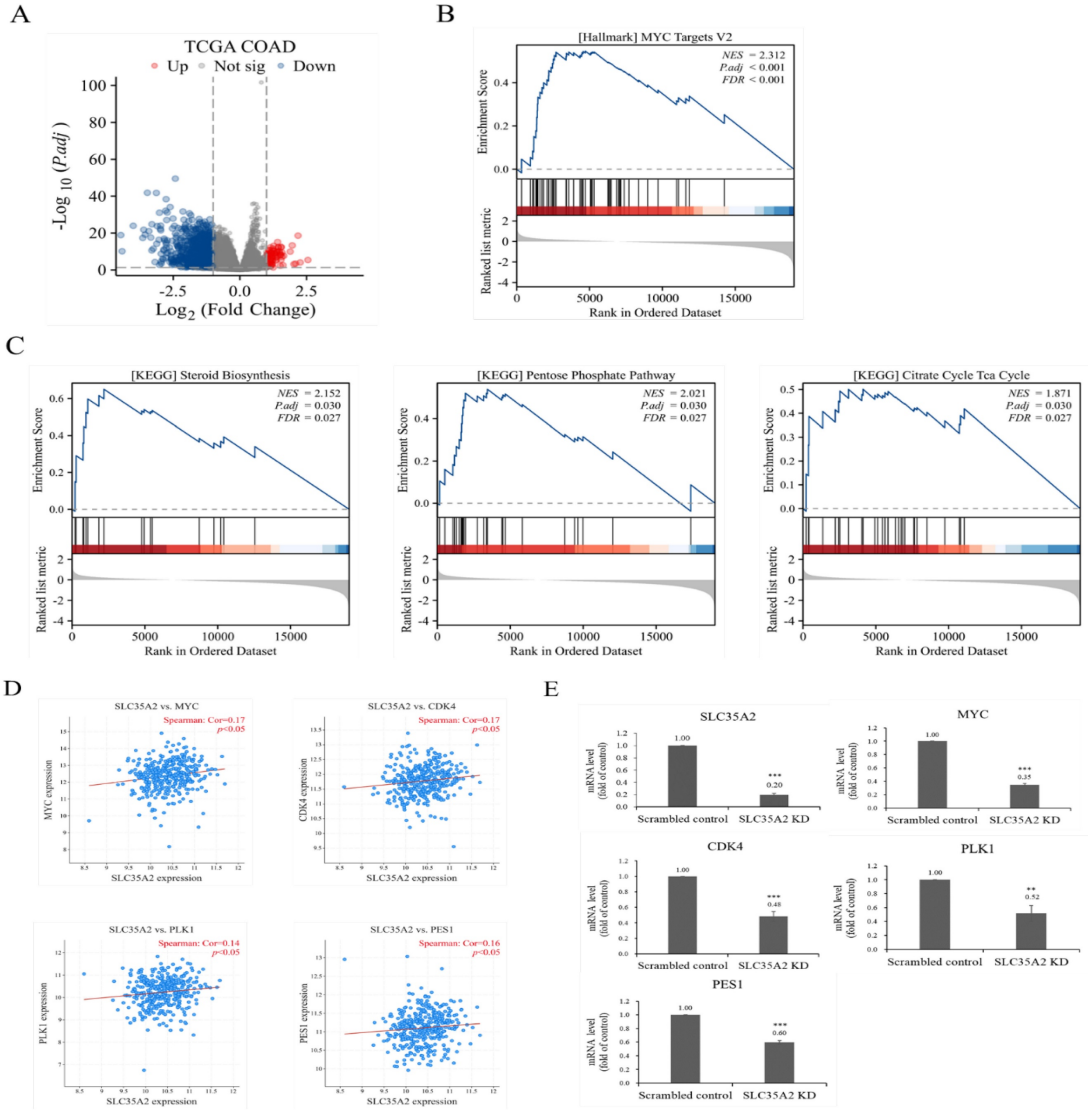
** SLC35A2 is associated with the MYC pathway in CRC.** (A) Volcano plot of differential expression genes between high (top 50%) - and low (bottom 50%) SLC35A2 expression derived from TCGA COAD data. GSEA analysis of (B) hallmark and (C) KEGG gene set collections using the log_2_ fold changes of DEGs. (D) Regression analysis between SLC35A2 and MYC-associated genes (MYC, CDK4, PLK1 and PES1) in CRC performed by cBioPortal. (E) RT-qPCR analysis of mRNA expression of SLC35A2 and MYC-associated genes (MYC, CDK4, PLK1 and PES1) in DLD-1 scrambled control and SLC35A2 KD cells.

Due to the aberrantly high expression of SLC35A2 in CRC and its association with poor patient prognosis, we further investigated the underlying mechanisms of SLC35A2 in CRC. Based on the data from TCGA COAD, we split patients into two groups, high and low SLC35A2 expression, based on the median expression level. Subsequently, differential expression analysis was performed between these two groups, resulting in a total of 1159 DEGs (Figure 5A). We performed GSEA analysis using the fold change of DEGs and obtained 15 significantly enriched pathways (Table S1). Among positive NES score pathways, hallmark gene sets analysis revealed MYC Targets V2 (Figure 5B). Kyoto Encyclopedia of Genes and Genomes (KEGG) gene sets analysis also identified Steroid Biosynthesis, Pentose Phosphate Pathway, and Citrate Cycle TCA Cycle (Figure 5C). These findings suggest that SLC35A2 may influence cancer cell metabolic reprogramming in CRC through downstream effectors of MYC. To clarify the relationship between SLC35A2 and MYC-associated genes, we performed a correlation analysis on clinical sample data from cBioPortal. The results showed that in colorectal cancer, the expression of SLC35A2 was positively correlated with the expression of four MYC-associated genes, including MYC, Cyclin dependent kinase 4 (CDK4), Polo like kinase 1 (PLK1), and Pescadillo ribosomal biogenesis factor 1 (PES1) (Figure 5D). Next, we knocked down SLC35A2 in DLD-1 cells and used RT-qPCR to examine the expression levels of SLC35A2 and MYC-associated genes. The results showed that after silencing SLC35A2, the expression of four MYC-associated genes was also downregulated (Figure 5E). The findings supported the role of SLC35A2 in regulating the MYC pathway in CRC.

### Silencing of SLC35A2 suppressed CRC cell proliferation and migration

**Figure 6:**
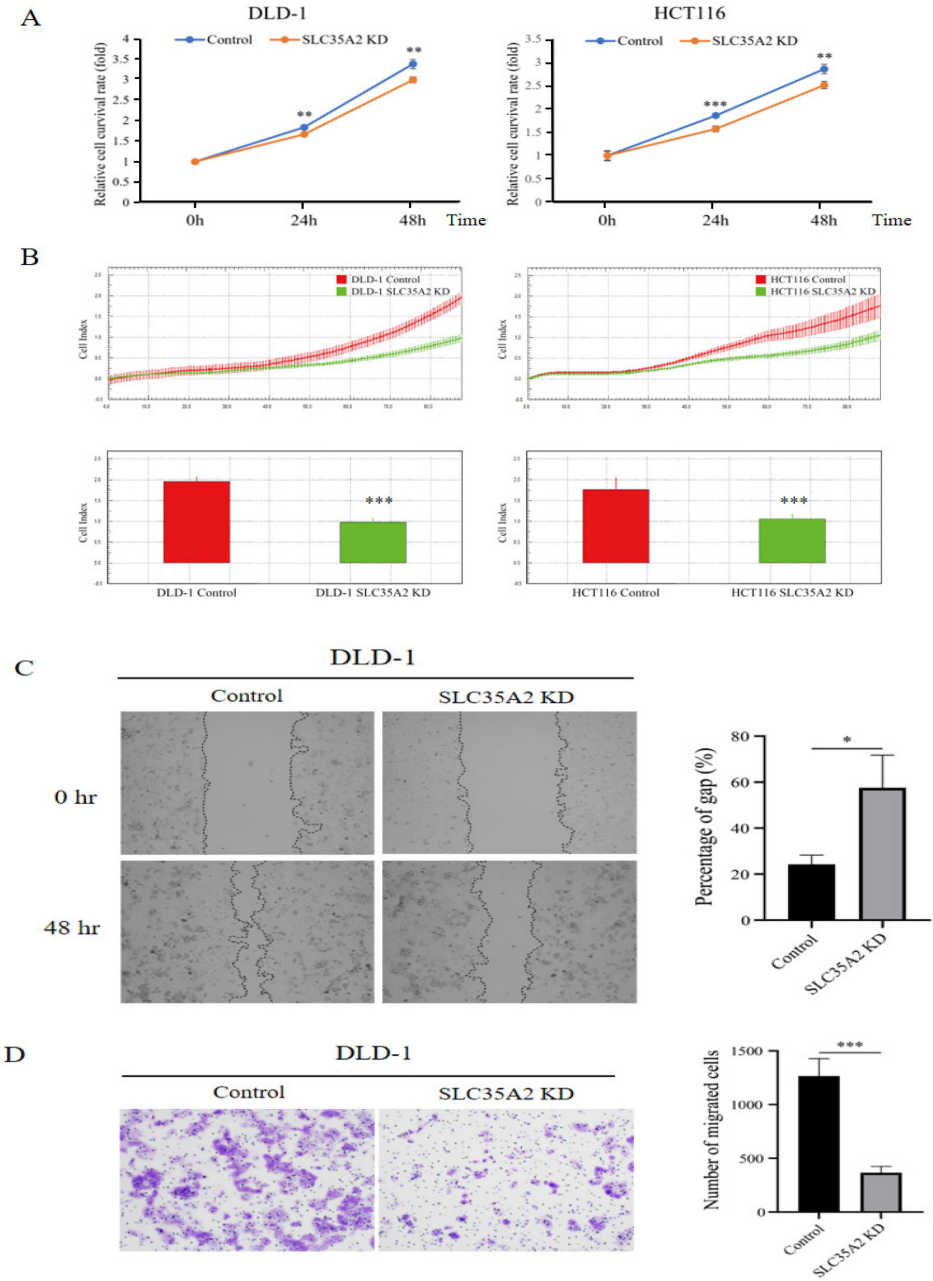
** SLC35A2 knockdown inhibited CRC cell proliferation and migration.** (A)SRB assays were performed to evaluate the proliferation of SLC35A2 knockdown DLD-1 and HCT 116 cells. (B) Representative real-time impedance measurement of the control and knockdown cells of DLD-1 and HCT 116 using the xCELLigence system were showen. (C) Wound healing assay and (D) transwell migration assay detected the migration ability of DLD-1 cells in the control and SLC35A2 knockdown group.

We discovered that SLC35A2 enhances MYC expression in CRC, and since MYC is a crucial transcription factor promoting colorectal cancer progression 2, this suggests a potential role for SLC35A2 in cancer development. To further investigate the function of SLC35A2 in CRC progression, we first knocked down SLC35A2 in the DLD-1 and HCT 116 cells (Supplementary Figure 2) and used SRB assays to assess cell proliferation. The results indicated that the SLC35A2-silenced group exhibited lower cell viability compared to the control group (Figure 6A). Additionally, we used the xCELLigence system to monitor cell proliferation, which further confirmed that after silencing SLC35A2, the cell index significantly decreased (Figure 6B). These findings indicate that SLC35A2 plays a role in promoting CRC cell proliferation. Since the metastatic ability of cancer cells is closely related to cancer progression 32, we next focused on evaluating whether SLC35A2 affects the migration ability of cancer cells. The results of the wound healing assay showed that after knocking down SLC35A2, the migration of DLD-1 cells was reduced (Figure 6C). Additionally, we performed a transwell migration assay, which indicated that fewer cells moved from the upper chamber to the lower chamber in SLC35A2-knockdown DLD-1 cells (Figure 6D). In summary, our data suggests that SLC35A2 promotes CRC cell proliferation and metastasis.

### Associations between SLC35A2 and chemotherapeutic drug sensitivity

**Figure 7:**
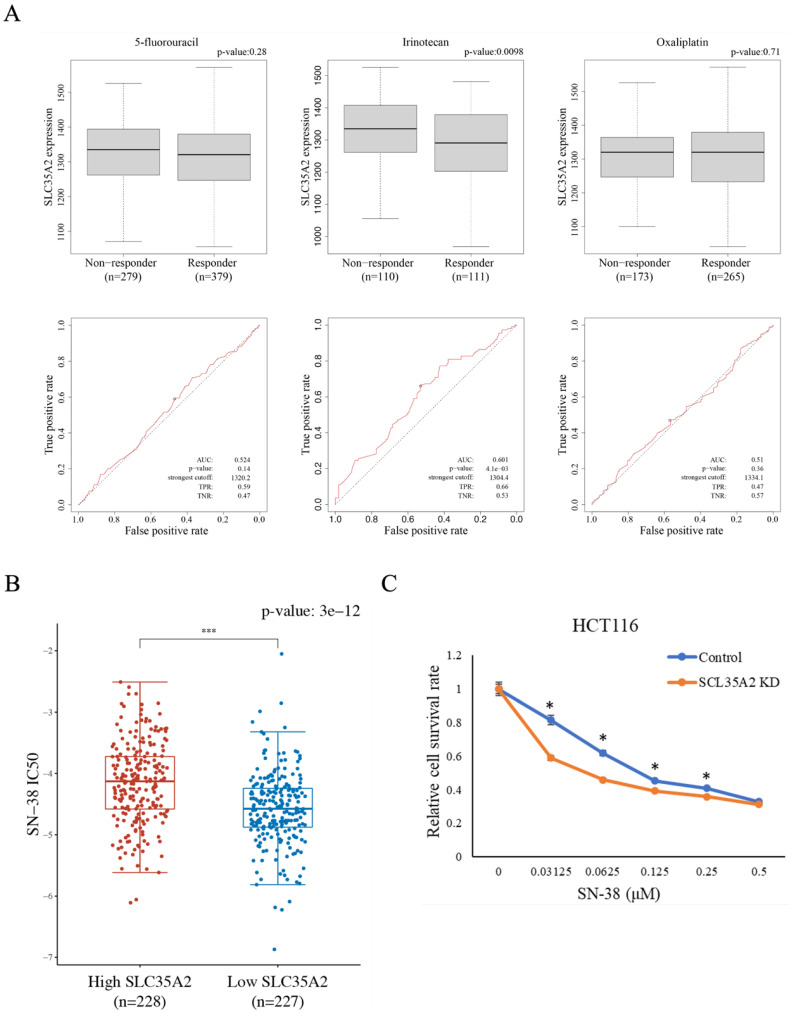
** Chemotherapeutic drug sensitivity analysis of SLC35A2 in CRC.** (A) Box plots were used to illustrate the expression of SLC35A2 in CRC patients stratified by chemotherapy responses according to the Response Evaluation Criteria in Solid Tumors (RECIST) criteria. Receiver operating characteristic (ROC) analysis was conducted to assess the predictive value of SLC35A2 expression for therapy response. (B) The IC_50_ of the high SLC35A2 expression group in response to SN-38 was higher than the low expression group. (C) Cell viability of HCT 116 cells after treatment with SN-38 for 48h. The cell viability data was obtained using the SRB assay. Viability data points were normalized to a concentration of 0 μM and expressed as a percentage. Three individual experiments are shown as means and standard deviations. AUC, the area under the curve; TPR, true positive rate; TNR, true negative rate.

CRC patients with high SLC35A2 expression have lower relapse-free survival than those with low SLC35A2 expression, suggesting a potential association between SLC35A2 and treatment response. Therefore, we analyzed the correlation between SLC35A2 expression and the chemotherapeutic response in CRC patients using the ROC plotter website. Patients were divided into two groups based on treatment response, and the expression levels of SLC35A2 were compared between the two groups (Figure 7A). The results revealed that patients with poor response to irinotecan exhibited higher expression of SLC35A2 compared to those with good response, while there was no significant difference in expression levels between the two groups for 5-fluorouracil and oxaliplatin. Furthermore, based on the ROC curve analysis, an increased expression of SLC35A2 could effectively distinguish non-responders from responders to irinotecan therapy (AUC = 0.601; p = 4.1e-03). To further validate the relationship between SLC35A2 and irinotecan sensitivity, we calculated the IC50 values for SN-38 (an active metabolite of irinotecan) in TCGA COAD samples based on the GDSC database. The results demonstrated that high expression of SLC35A2 was significantly associated with higher IC_50_ values compared to low expression (Figure 7B). Next, we knocked down SLC35A2 in HCT 116 cells and used the SRB assay to examine the viability of control and knockdown cells under different concentrations of SN-38. The results showed that SLC35A2-knockdown cells had significantly increased sensitivity to SN-38 (Figure 7C). These findings indicate that SLC35A2 plays an important role in the mechanism of irinotecan resistance in CRC.

### Relationship between SLC35A family expression and immune infiltration in CRC

**Figure 8:**
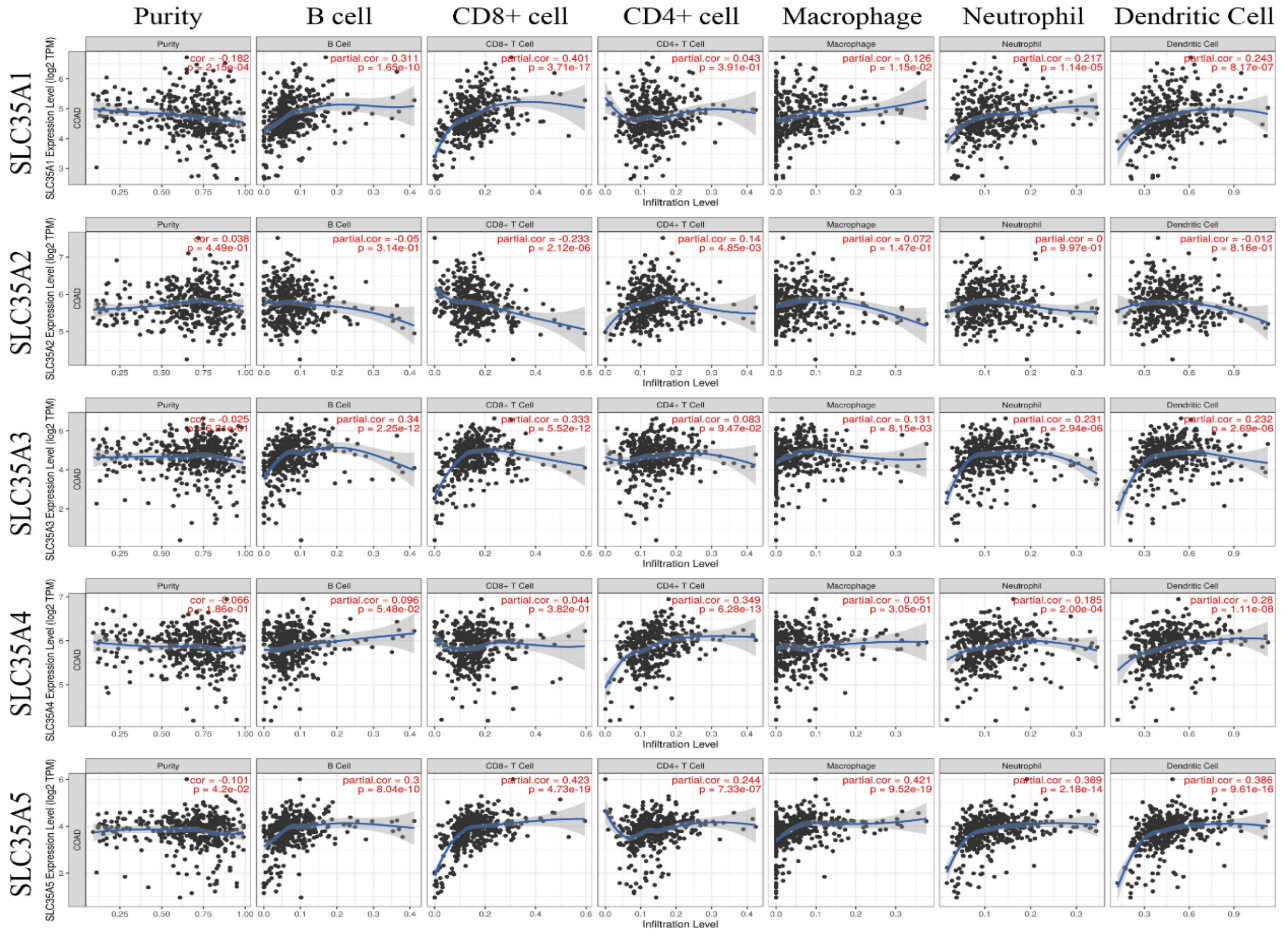
Correlation of SLC35A1-5 expression with the level of immune infiltration in COAD.

Many studies have emphasized the significance of tumor-infiltrating immune cells in the prognosis and therapeutic efficacy of CRC 33. Therefore, we employed the TIMER database to investigate the relationship between SLC35A family genes and immune cell infiltration (Figure 8). The results revealed that the expressions of SLC35A1 and SLC35A5 were negatively correlated with tumor purity (p < 0.05). The expression of SLC35A5 showed a positive correlation with the infiltration of six types of immune cells, including B cells, CD8+ T cells, CD4+ T cells, macrophages, neutrophils, and dendritic cells. On the other hand, the expression levels of SLC35A1 and SLC35A3 were positively correlated with the infiltration of B cells, CD8+ T cells, macrophages, neutrophils, and dendritic cells. Furthermore, the expression of SLC35A4 was positively correlated with the infiltration of CD4+ T cells, neutrophils, and dendritic cells. It is worth noting that although the expression of SLC35A2 was positively correlated with CD4+ T cells, it exhibited a negative correlation with CD8+ T cells (P < 0.001, r = -0.233). We also found that SLC35A2 expression negatively correlates with effector memory CD8+ T cells infiltration (rho= -0.305, P < 0.001) and patients with high SLC35A2 expression and low memory CD8+ T cells have poor survival. In addition to that, SLC35A2 also negatively correlates with activated B cell infiltration (rho= -0.22, P < 0.001) and patients with high SLC35A2 expression and low B cells have poor survival (Supplementary Figure 3).

### Relationship between SLC35A family expression and immune infiltration in CRC

Many studies have emphasized the significance of tumor-infiltrating immune cells in the prognosis and therapeutic efficacy of CRC 33. Therefore, we employed the TIMER database to investigate the relationship between SLC35A family genes and immune cell infiltration (Figure 8). The results revealed that the expressions of SLC35A1 and SLC35A5 were negatively correlated with tumor purity (p < 0.05). The expression of SLC35A5 showed a positive correlation with the infiltration of six types of immune cells, including B cells, CD8+ T cells, CD4+ T cells, macrophages, neutrophils, and dendritic cells. On the other hand, the expression levels of SLC35A1 and SLC35A3 were positively correlated with the infiltration of B cells, CD8+ T cells, macrophages, neutrophils, and dendritic cells. Furthermore, the expression of SLC35A4 was positively correlated with the infiltration of CD4+ T cells, neutrophils, and dendritic cells. It is worth noting that although the expression of SLC35A2 was positively correlated with CD4+ T cells, it exhibited a negative correlation with CD8+ T cells (P < 0.001, r = -0.233). We also found that SLC35A2 expression negatively correlates with effector memory CD8+ T cells infiltration (rho= -0.305, P < 0.001) and patients with high SLC35A2 expression and low memory CD8+ T cells have poor survival. In addition to that, SLC35A2 also negatively correlates with activated B cell infiltration (rho= -0.22, P < 0.001) and patients with high SLC35A2 expression and low B cells have poor survival (Supplementary Figure 3).

## Discussion

The current study observed that SLC35A2 exhibits higher expression in CRC tumor tissues than in normal tissues. Furthermore, patients with high SLC35A2 expression showed a poorer prognosis. We performed the GSEA analysis to investigate the pathways of SLC35A2 using TCGA data. The results revealed that SLC35A2 might influence cancer cell metabolism through downstream molecules related to MYC, including pathways such as steroid biosynthesis, pentose phosphate pathway, and tricarboxylic acid (TCA) cycle. Cancer cells employ several metabolic pathways, including glucose, amino acid, and lipid metabolism, to sustain their high cell division rates 34. The pentose phosphate pathway (PPP) is a crucial branch of glucose metabolism that diverges from glycolysis 35. It is essential for nucleotide synthesis and is a primary nicotinamide adenine dinucleotide phosphate (NADPH) source. NADPH is required for intracellular reductive synthesis reactions like fatty acid synthesis and steroid biosynthesis, which fulfill energy demands of cancer proliferation 36. PPP and NADPH also play a vital role in the clearance of reactive oxygen species (ROS), while the metabolic reprogramming is critical in helping glycolytic cancer cells counteract oxidative stress 37. Recent studies have indicated that pharmacological inhibition of the mTOR-PPP axis holds promising potential as a therapeutic strategy against colorectal cancer 38. Further, MYC was reported to have the ability of regulating biogenesis and function of mitochondria, which increases production of acetyl-CoA during cell cycle as well as supplies substrates for histone acetylation 39. These MYC-regulated substrate increase in TCA cycle and metabolic pathways provide energy for cells, promoting tumor cell proliferation. Our results identify that these cancer metabolism-related gene sets and signaling pathways are closely related to SLC35A2-regulated MYC, indicating that SLC35A2-MYC holds an important role in CRC progression.

Our findings demonstrate a negative correlation between the expression of SLC35A2 and the sensitivity to irinotecan. Irinotecan is a commonly used chemotherapeutic agent for CRC. However, developing irinotecan resistance remains a major clinical challenge 40. The molecular mechanisms underlying irinotecan resistance are complex and involve interplay among multiple signaling pathways 41. The mechanism by which SLC35A2 causes drug resistance requires further study, yet the current research suggests the potential of SLC35A2 as a clinical therapeutic target or provides a hint of combined treatment avenue.

This study also shows that SLC35A2 negatively correlates with the infiltration of effector memory CD8+ T cells and activated B cells. Tumor-infiltrating immune cells play a crucial role in the development and progression of cancer and hold promise as prognostic markers 42. Cytotoxic CD8+ T cell has long been used in clinical therapy for cancers, while B cell also provides anti-tumor immunity 43. Tumor develops discrete mechanism to avoid the infiltration of these cytotoxic T cells 44. MYC can regulate multiple metabolic pathways in cancer cells and play a role directly or indirectly impacting tumor microenvironment and evading the host's immunity via its effects on metabolism 45, 46. These reports are consistent with our findings, indicating that SLC35A2-mediated MYC may affect cell metabolic pathways and further lead to immune elimination. Despite immunotherapy has shown promise in treating several cancers in past years, our study revealed that SLC35A2 is highly expressed in microsatellite stable (MSS) colorectal tumors, which is in consistent with unresponsiveness to the immunotherapy 47. Therefore, targeting SLC35A2 and MYC may be effective approaches for treating colorectal cancer. Nervertheless, although candidate drugs targeting MYC are under development, no drug has been proven to be effective in treating MYC or MYC-related pathways to date. The new target SLC35A2 is even unknown, which deserves further exploration and development.

SLC35A2 encodes a protein called UDP-galactose transporter, primarily located in the Golgi apparatus. It is responsible for transporting UDP-galactose from the cytoplasm to the Golgi vesicles, which are used for glycan synthesis 48. Mutations in the SLC35A2 gene lead to a rare congenital disorder of glycosylation known as SLC35A2-CDG. This disorder is characterized by features such as epileptic encephalopathy and cerebral palsy 49. The regulation of SLC35A2 in glycosylation also participates in the involvement of cancer process. SLC35A2 was reported to regulate cellular glycosylation modification and induce the adhesive activity of HCC cells, contributing to metastasis. The study suggests that the maintenance of SLC35A2 activity is crucial for recruiting the key galactosyltransferase B4GalT1, responsible for complex glycan assembly and lactose biosynthesis, into the Golgi apparatus of HCC cells 14. In breast cancer, a few studies have slightly explored the possible mechanism of SLC35A2. Yang et al. revealed SLC35A2 fosters breast cancer progression through activating extracellular signal regulated kinase (ERK) pathway 50. Another research identified high expression of SLC35A2 is positively correlated with overexpression of human epidermal growth factor receptor 2 (HER2) 51, which is associated with stimulation of the phosphoinositide 3-kinase (PI3K) / AKT and mitogen activated protein kinase (MAPK) sigmaling 52. Similary, Luo et al. revealed that SLC35A2 regulates mitochondrial autophagy in osteosarcoma cells via the PI3K/AKT/mTOR signaling pathway 53. Nevertheless, the role of SLC35A2 is not yet clearly identified in colorectal cancer. One bioinformatic analysis exhibited high expression of SLC35A2 in many cancer types including colon adenocarcinoma, and overexpression of SLC35A2 is related to decreased lymphocyte infiltration 15. Another research revealed that upregulation of SLC35A2 is positively correlated with lymph node metastasis and clinicopathological staging 54. These studies not only reveal the importance of SLC35A2 in regulation of cancer pathogenesis, but arouse the interests of researchers to perform a deeper exploration.

SLC35A1, located in the membrane of the Golgi apparatus, plays a crucial role in the transportation of nucleotide sugars, including cytidine 5'-monophosphate (CMP)-sialic acid, to the Golgi apparatus. Once inside the Golgi, SLC35A1 facilitates the glycosylation of CMP-sialic acid, thereby participating in the sialylation 55. Some studies have pointed out defects in the SLC35A1 gene are responsible for developing Congenital Disorder of Glycosylation type 2F (CDG2F) 56. Our study showed downregulation of SLC35A1 expression in various tumors, including colorectal cancer, compared to adjacent normal tissues. Additionally, we found a higher degree of DNA methylation in the SLC35A1 promoter region in tumor tissues, suggesting that the altered expression of SLC35A1 may be associated with abnormal methylation patterns. Furthermore, we identified a significant positive correlation between SLC35A1 expression and infiltration of CD8+ T cells, indicating that SLC35A1 may influence immune cell infiltration through tumor cell surface sialylation. Elevating sialylation level on tumor cell surfaces is often associated with tumor invasion and poor prognosis in malignant tumors 57. Studies have indicated that increased sialylation in B16 melanoma inhibits the formation of effector T cells and promotes the presence of regulatory T cells (Tregs), significantly affecting tumor growth 58. Therefore, it is worthwhile to investigate how the altered salivary acidification caused by SLC35A1 affects immune cell activity in the tumor microenvironment.

SLC35A3 encodes a transporter located in the Golgi membrane which transports uridine diphosphate (UDP)-N-acetylglucosamine from the cytoplasm to the Golgi vesicles as a sugar donor for oligosaccharide synthesis 59. We found that SLC35A3 is generally overexpressed in tumors compared to normal tissues. Studies have indicated that SLC35A3 is aberrantly upregulated in pancreatic ductal carcinoma and affects glycolysis, which is also associated with patient prognosis 60. Our results revealed that SLC35A3 is downregulated in CRC compared to adjacent normal tissue. However, the DNA methylation level of SLC35A3 in CRC is lower than that in adjacent tissue, suggesting that some cancer-related transcriptional regulations may influence the gene expression of SLC35A3 during tumorigenesis. SLC35A3 is associated with immune cell infiltration in CRC, including B cells, CD8+ T cells, macrophages, neutrophils, and dendritic cells. In breast cancer, Ta et al. found a correlation between SLC35A3 expression and immune cell infiltration, suggesting that SLC35A3, along with CD8+ T cells, could serve as a biomarker for assessing immunotherapy efficacy 13.

SLC35A4 encodes a putative nucleotide sugar transporter. Recent studies suggest that its role may not be related to glycosylation directly but rather involved in the regulation or modulation of UDP-galactose transporter (UGT; SLC35A2) and UDP-N-acetylglucosamine transporter (NGT; SLC35A3) activities 8. Furthermore, research indicates that SLC35A4 does not form homodimers and is directly associated with another putative UDP sugar transporter, orphan SLC35A5 61. The role of the SLC35A5 in large-scale glycosylation remains unclear. Studies have shown that the inactivation of the SLC35A5 gene leads to impaired uptake of UDP-glucuronic acid, UDP-N-acetylglucosamine, and UDP-N-acetylgalactosamine into the Golgi apparatus while not affecting UDP-galactose transport activity 12. In our study, we observed that the expression of SLC35A4 and SLC35A5 is decreased in colorectal cancer compared to adjacent non-cancerous tissues. Furthermore, their expression levels were positively correlated with immune cell infiltration. However, no significant association was found between the expression of these two genes and patient prognosis. It is worth noting that previous research has indicated the involvement of SLC31A2, SLC43A1, SLC35A5, and SLC41A2 in paclitaxel sensitivity, as well as their role in modulating single-nucleotide polymorphism (SNPs) associated with paclitaxel-induced cytotoxicity 62.

The metastatic disease occurs in approximately 20% of patients with colorectal cancer 63. Although surgery, chemotherapy, radiation therapy, and targeted drugs are available for CRC 64, 65, inoperable metastatic colorectal cancer (mCRC) remains a life-threatening condition 66. Dysregulation of SLCs in cancer cells leads to metabolic reprogramming, playing a crucial physiological role in tumor initiation, progression, and treatment resistance. Modulating the activity of SLCs represents a potential therapeutic strategy 67. The functional role of SLC35A family genes in CRC is still unclear, and more studies are needed to explore their therapeutic applications. This study employs integrative bioinformatics approaches to investigate the role of the SLC35A family in CRC and utilizes cell models to verify the oncogenic role of SLC35A2. Future validation studies involving clinical CRC samples are necessary to elucidate the feasibility of utilizing SLC35A2 to diagnose or treat CRC. The current literature on the role of SLC35A family members in CRC is limited. Our study provides valuable information that can help advance our understanding of the role of SLCs in CRC.

## Conclusions

Our study showed that CRC patients with high expression of SLC35A2 had lower relapse-free survival than those with low expression. In addition, we found that SLC35A2 plays an essential role in MYC-mediated metabolic reprogramming, and it is associated with irinotecan sensitivity. Moreover, a high SLC35A2 expression level might determine the immune infiltration of cytotoxic CD8+ T cells and B cells. Thus, high SLC35A2 levels in microsatellite stable (MSS) tumors might be responsible for the failure of immunotherapy in colorectal cancers. In conclusion, SLC35A2 could be a potential prognostic biomarker and therapeutic target for CRC.

## Supplementary Material

Supplementary figures and table.

## Figures and Tables

**Table 1:** Univariate and multivariate Cox regression model for progression-free interval in patients with COAD in TCGA.
