# Supplementary material for: Solute Carrier Family 35 A2 (SLC35A2) Promotes Tumor Progression through MYC-Mediated Pathways in Colorectal Cancer
Source: Int J Med Sci. 2025 Mar 29;22(9):1992–2009. doi: 10.7150/ijms.109767 (PMC12035830; doi:10.7150/ijms.109767)
Supplement: Supplementary file 1 — Supplementary figures and table. [file ijmsv22p1992s1.pdf]

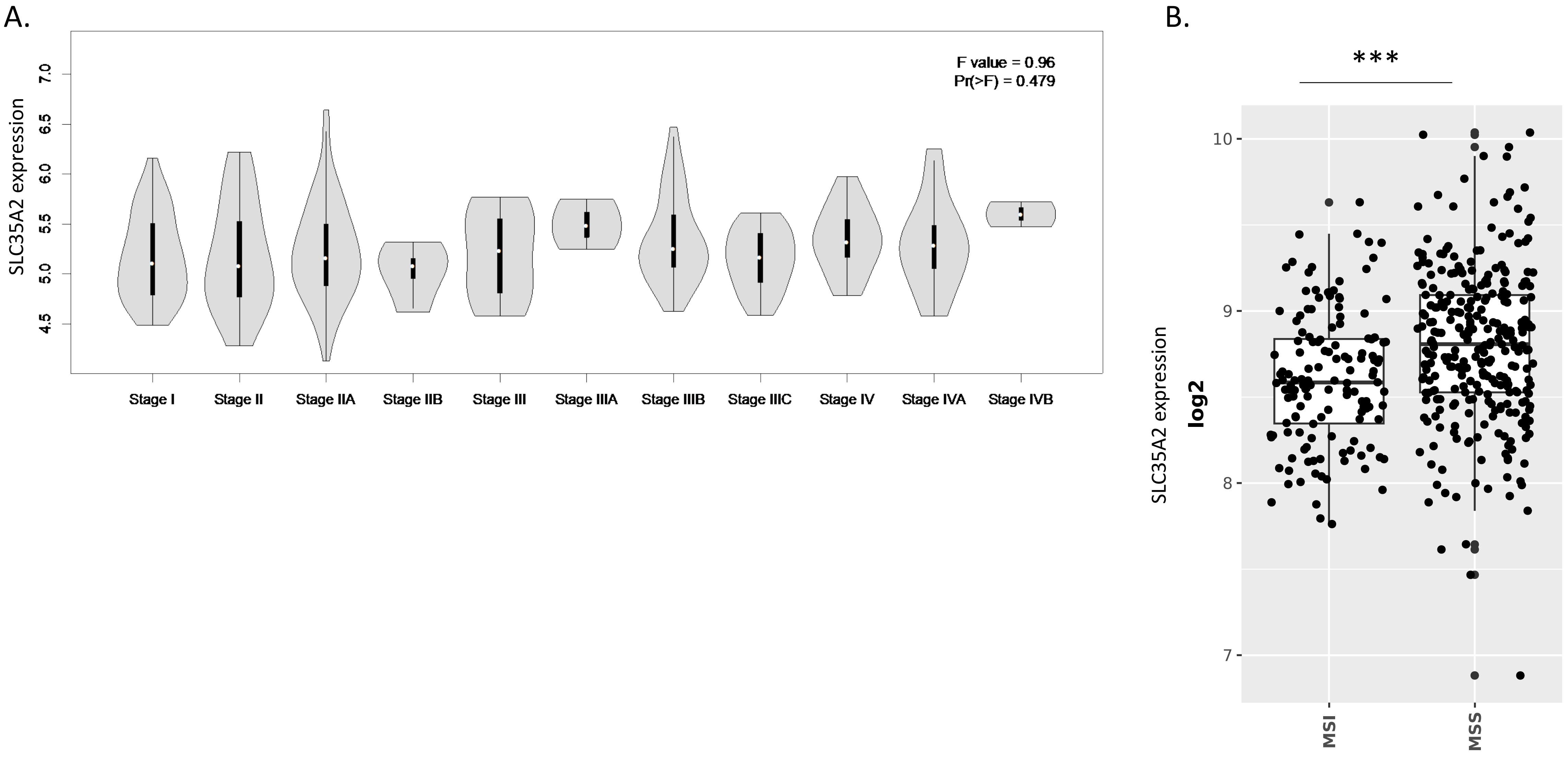

Figure S1. SLC35A2 expression is enhanced in advanced CRC. A. SLC35A2 expression levels across different tumor stage by GEPIA portal. B. SLC35A2 expression level based on microsatellite stability in CRC by GENT2 portal. MSS: microsatellite stable; MSI: microsatellite instable. *** p=<0.001.

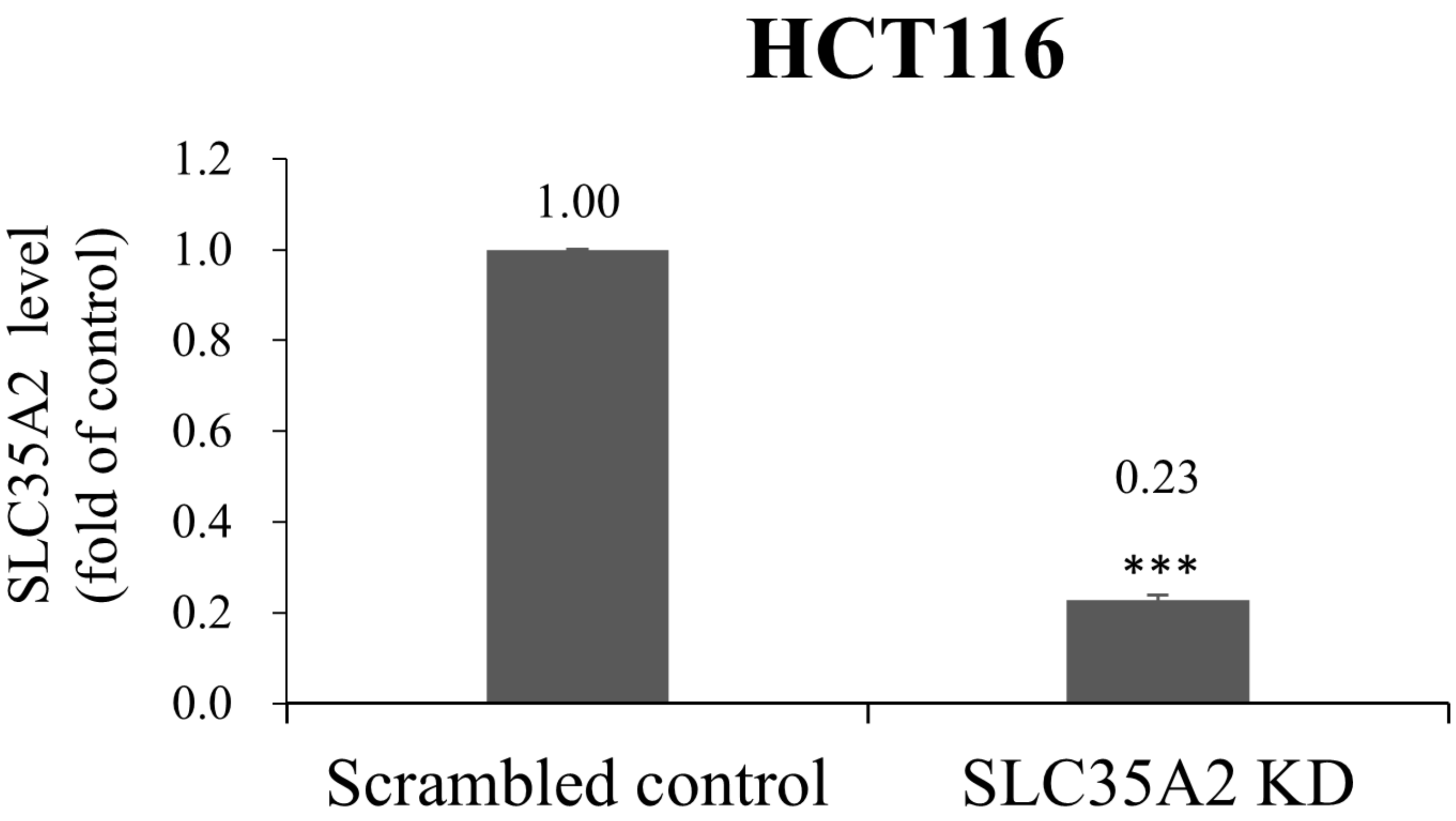

Figure S2. The confirmation experiment of SLC35A2 knockdown in HCT 116 cells. The confirmation test of SLC35A2-KD in DLD-1 cells had already been presented in Figure 5E. *** p=<0.001.

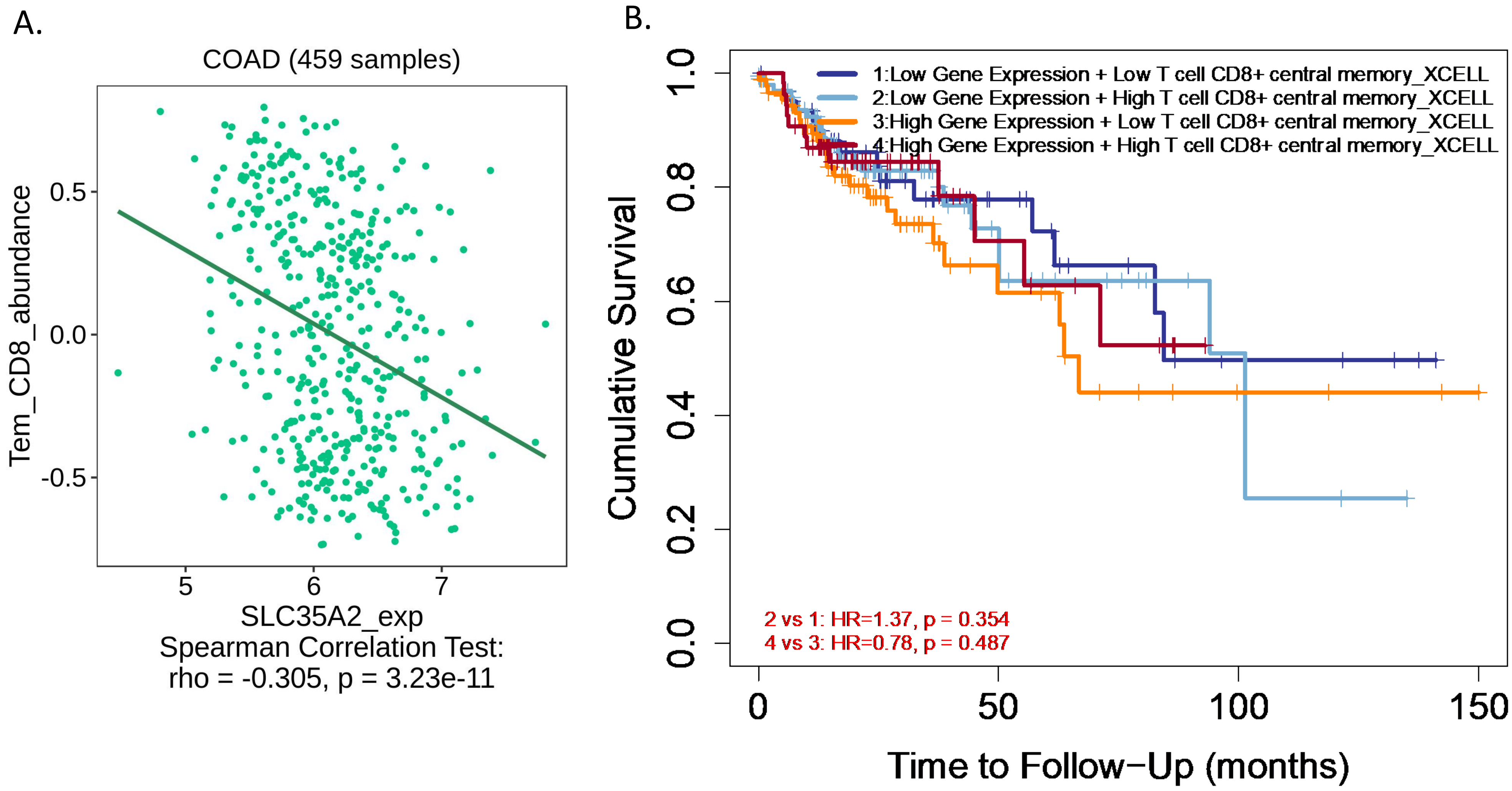

Figure S3. SLC35A2 correlates to low infiltration of B cell and CD8+ T cells. A. Spearman Correlation of CD8+ T cell and SLC35A2 expression levels by TISIDB portal. B. Cumulative survival plot of High/low SLC35A2 with High/low CD8+ T cell infiltration in COAD by TIMER 2.0 portal.

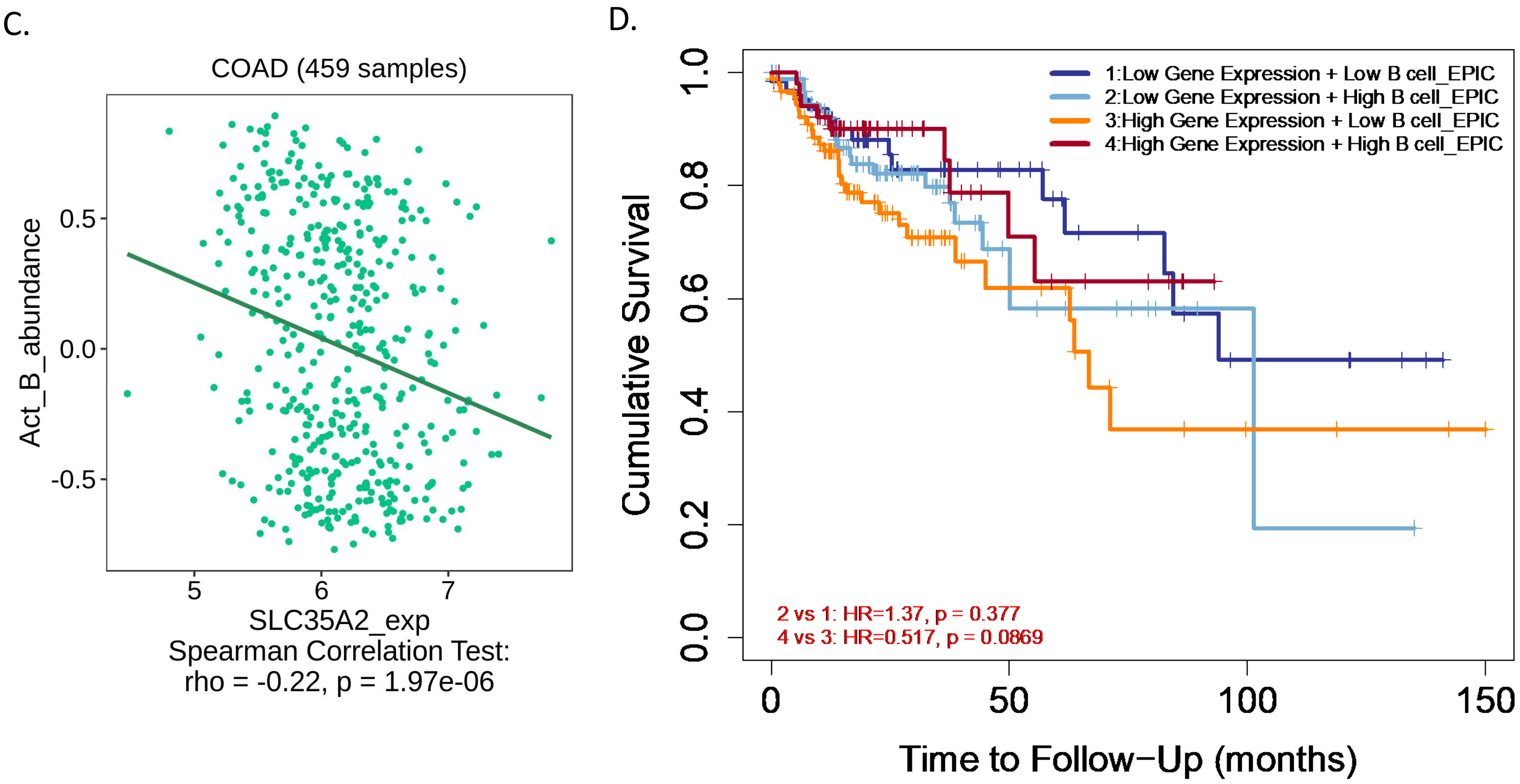

Figure S3. SLC35A2 correlates to low infiltration of B cell and CD8+ T cells. C. Spearman Correlation of activated B cell and SLC35A2 expression levels by TISIDB portal. D. Cumulative survival plot of High/low SLC35A2 with High/low B cell infiltration in COAD by TIMER 2.0 portal.

Table S1 GSEA results of DEGs.

**Pathway ID**	**enrichmentScore**	**NES**	**p.adjust**	**qvalue**
KEGG_STEROID_BIOSYNTHESIS	0.65	2.15	3.05E-02	2.72E-02
KEGG_PENTOSE_PHOSPHATE_PATHWAY	0.54	2.02	3.05E-02	2.72E-02
KEGG_CITRATE_CYCLE_TCA_CYCLE	0.50	1.87	3.05E-02	2.72E-02
KEGG_CYTOKINE_CYTOKINE_RECEPTOR_INTERACTION	-0.48	-1.34	1.39E-02	1.24E-02
KEGG_CALCIUM_SIGNALING_PATHWAY	-0.51	-1.41	5.42E-03	4.83E-03
KEGG_HEMATOPOIETIC_CELL_LINEAGE	-0.54	-1.47	3.05E-02	2.72E-02
KEGG_GRAFT_VERSUS_HOST_DISEASE	-0.63	-1.59	3.14E-02	2.81E-02
KEGG_NEUROACTIVE_LIGAND_RECEPTOR_INTERACTION	-0.59	-1.66	5.90E-09	5.26E-09
KEGG_SYSTEMIC_LUPUS_ERYTHEMATOSUS	-0.65	-1.80	5.90E-09	5.26E-09
KEGG_OLFACTORY_TRANSDUCTION	-0.65	-1.82	5.90E-09	5.26E-09
KEGG_TASTE_TRANSDUCTION	-0.70	-1.82	2.10E-05	1.88E-05
HALLMARK_MYC_TARGETS_V2	0.55	2.31	4.77E-06	4.18E-06
HALLMARK_EPITHELIAL_MESENCHYMAL_TRANSITION	-0.49	-1.35	1.32E-02	1.16E-02
HALLMARK_INFLAMMATORY_RESPONSE	-0.49	-1.37	9.81E-03	8.60E-03
HALLMARK_ALLOGRAFT_REJECTION	-0.50	-1.39	8.85E-03	7.76E-03
